# Control Growth Factor Release Using a Self-Assembled [polycation∶heparin] Complex

**DOI:** 10.1371/journal.pone.0011017

**Published:** 2010-06-08

**Authors:** Blaine J. Zern, Hunghao Chu, Yadong Wang

**Affiliations:** Wallace H. Coulter Department of Biomedical Engineering, School of Chemistry and Biochemistry, and Institute for Bioengineering and Biosciences, Georgia Institute of Technology, Atlanta, Georgia, United States of America; Massachusetts Institute of Technology, United States of America

## Abstract

The importance of growth factors has been recognized for over five decades; however their utilization in medicine has yet to be fully realized. This is because free growth factors have short half-lives in plasma, making direct injection inefficient. Many growth factors are anchored and protected by sulfated glycosaminoglycans in the body. We set out to explore the use of heparin, a well-characterized sulfated glycosaminoglycan, for the controlled release of fibroblast growth factor-2 (FGF-2). Heparin binds a multitude of growth factors and maintains their bioactivity for an extended period of time. We used a biocompatible polycation to precipitate out the [heparin∶FGF-2] complex from neutral buffer to form a release matrix. We can control the release rate of FGF-2 from the resultant matrix by altering the molecular weight of the polycation. The FGF-2 released from the delivery complex maintained its bioactivity and initiated cellular responses that were at least as potent as fresh bolus FGF-2 and fresh heparin stabilized FGF-2. This new delivery platform is not limited to FGF-2 but applicable to the large family of heparin-binding growth factors.

## Introduction

**Figure 1:**
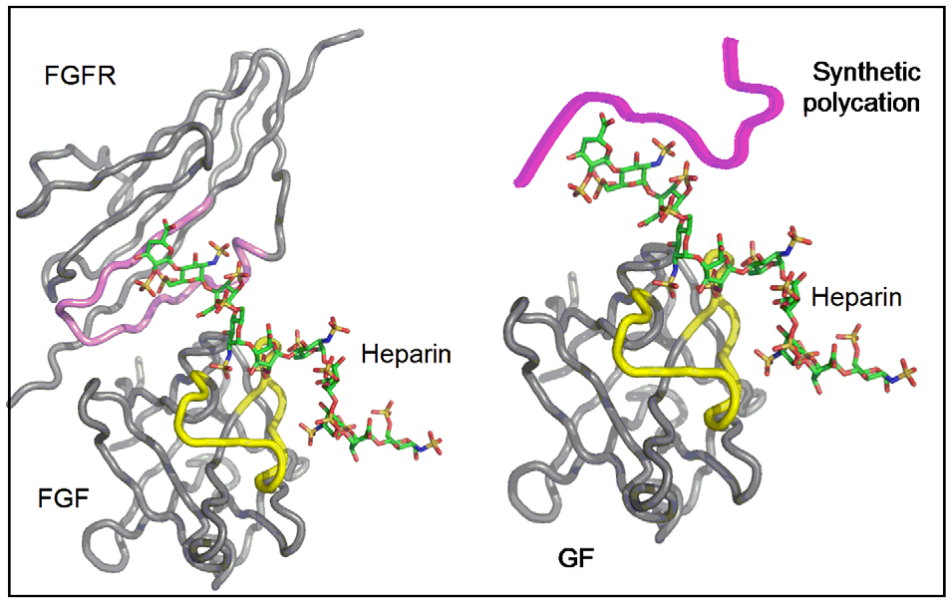
Our delivery strategy was inspired by the interaction among growth factor, heparin and growth factor receptor. *Left*, the crystal structure of the [FGF∶Heparin∶FGFR] complex kindly provided by Dr. Pellegrini. The proteins are shown as coils and heparin as a stick model. The heparin-binding domains of FGFR and FGF are highlighted in pink and yellow respectively. Both analyses showed that the heparin-binding regions contain a high density of positively charged amino acid residues such as arginine. *Right*, a possible model of the matrix formed by ionic interactions between an arginine-based synthetic polycation and a [heparin∶growth factor] complex.

Growth factors play an important role in regulating cell activity and body function [1]–[3]. The short half-life of free growth factors in plasma and potential side effects of systemic delivery indicate that a controlled delivery strategy will be needed to achieve high therapeutic efficacy [4]–[10]. Naturally occurring glycosaminoglycans, such as heparin, bind and stabilize many growth factors, and at least in FGF-2, present it in a more bioactive form (**Fig. 1** **Left**) [11]–[14]. We reasoned that a biocompatible and biodegradable polycation could bind heparin polyvalently and precipitate out the [heparin∶growth factor] complex by charge neutralization. The use of intact heparin is inspired by the ternary structure of [FGF-2∶heparin∶FGF-2 receptor] (**Fig. 1**). The arginine-based polycation, poly(argininate glyceryl succinate) (PAGS) is a minimalistic mimetic of the cationic domain of the FGF-2 receptor. To the best of our knowledge, this is the first report on controlled release of growth factors using a self-assembled [polycation∶heparin] matrix. We discovered that the simple, self-assembled matrix formed by PAGS, heparin, and FGF-2 in an aqueous buffer released bioactive growth factor at a rate controlled by PAGS's molecular weight. Not only can this matrix control the FGF-2 release kinetics but the released FGF-2 also possesses higher bioactivity than fresh bolus FGF-2, and equals that of fresh heparin protected FGF-2. Because heparin binds a broad spectrum of growth factors, this delivery platform is expected to offer controlled release of many growth factors with retention of bioactivity.

Growth factors are typically administered by bolus injections. Although therapeutic effects may be observed in animal models [15], [16], many of the simple injection regimens reported no significant improvement in phase II clinical trials [17], [18]. This has motivated a tremendous effort in controlled delivery of growth factors, which typically uses one of the following approaches: 1. Hydrogels [19]–[22], 2. polymer microparticles and films [23], [24], and 3. direct embedding of the growth factors in the delivery matrix [25], [26]. A large variety of biomaterials have been employed as delivery vehicles including alginate-, chitosan-, fibrin-, hyaluronan- and poly(ethylene glycol)-based hydrogels, collagen, acrylic acid derivatives, poly(α-hydroxyl acid)s, and inorganic salts such as calcium phosphate [27]–[30]. Heparin has been used in growth factor delivery systems. For example, heparin-coated alginate gels was used to deliver FGF-2 [31]. Heparin crosslinked with poly(lactide-co-glycolide) (PLG) [32] and collagen gel were used to deliver VEGF and FGF-2 [33]. Relatively few studies have used intact heparin as an integral component of a growth factor delivery vehicle, thus not taking full advantage of the enhancement of heparin on growth factor potency. In the existing studies using intact heparin, heparin-binding peptide epitopes were used to yield the delivery vehicle. One used multi-arm poly(ethylene glycol) modified with heparin-binding peptides (12 to 17mer) [34]. To the best of our knowledge, growth factor release has yet to be reported using this approach. Another recent report described the use of a heparin-binding peptide amphiphile to deliver VEGF and FGF-2 [35]. The delivery vehicle required 15-mer peptides and released 50% of the growth factors within 10 days *in vitro*. Both of these systems required expensive peptides and it is not reported how the growth factor release will be controlled. To the best of our knowledge, controlling growth factor release by varying the polyvalency between a simple polycation and heparin is unexplored.

## Results

**Figure 2:**
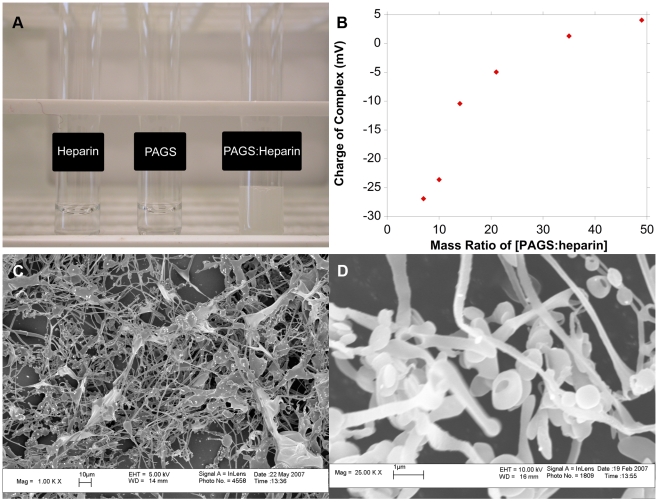
The interaction between PAGS and heparin. [A] The binding of PAGS and heparin resulted in a white precipitate when combined in an aqueous solution. [B] The titration of heparin using PAGS as monitored by zeta potential measurements. The complex was nearly neutral at a 35/1 mass ratio of PAGS/heparin. [C] SEM images revealed the [PAGS∶heparin] complexes as a matrix composed of fibers and sheets, and beads (1000×). [D] Higher magnification (25000×) revealed that many of the fiber are sub-micron in diameter and the beads were in fact rings.

The self-assembly of the delivery matrix from PAGS and heparin occurred in aqueous media with a white precipitate formed immediately upon mixing of the two solutions (**Fig. 2a**). The [PAGS∶heparin] complex was a suspension and can be pelleted by centrifugation. The charge of [PAGS∶heparin] complexes was examined via zeta potential titration by adding a PAGS solution into a heparin solution (**Fig. 2b**). A solution of heparin alone was approximately −30 mV. As PAGS solution was added, the zeta potential increased following a sigmoidal curve. A molar ratio of approximately 6/1 (mass ratio 35/1) of PAGS to heparin resulted in an almost neutral complex. This ratio was used for subsequent experiments because we expected it to afford the highest FGF-2 loading. Scanning electron microscopy (SEM) was used to further characterize the delivery matrix. SEM revealed [PAGS∶heparin] complexes as a matrix composed of fibers, sheets, and beads (**Fig. 2c**). Fiber diameters were approximately 1 µm to sub-micron and the sheets ranged from 5–20 µm in dimension. Examination of the matrix at 25000× magnification (**Fig. 2d**) revealed the fibers were more like thin ribbons and the beads were actually rings 0.5–1 µm in diameter.

**Figure 3:**
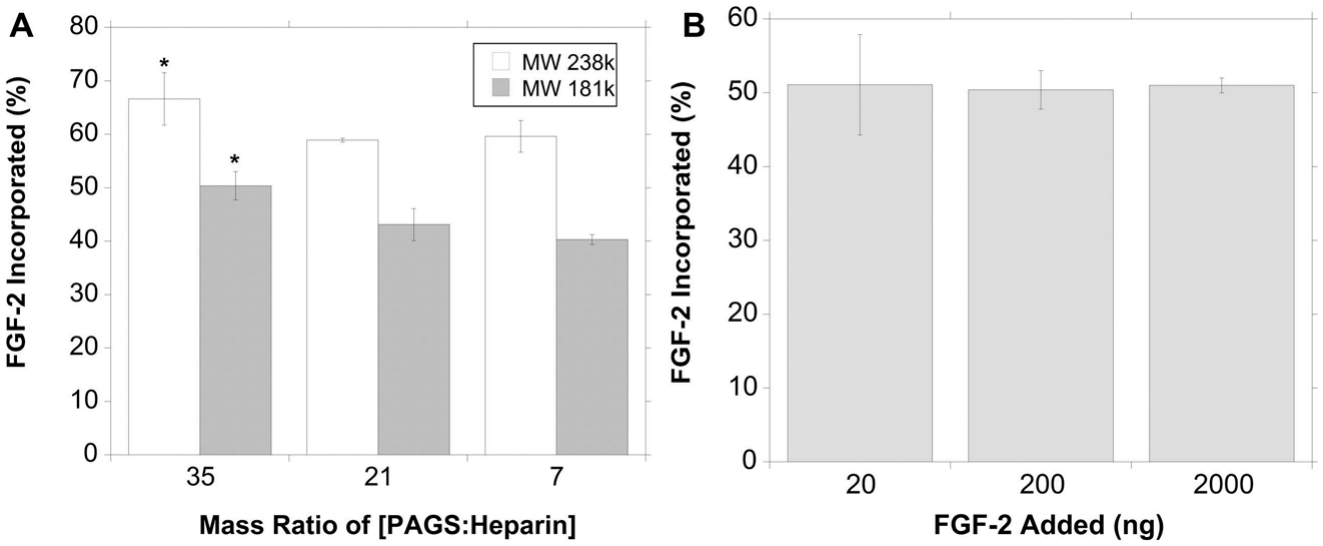
Complex loading efficiency and capacity was investigated using ^125^I-FGF-2. [A] Loading efficiencies of different molecular weight PAGS was investigated for different [PAGS∶heparin] ratios. The higher molecular weight PAGS was more efficient at incorporating FGF-2 at all [PAGS∶heparin] ratios than the lower molecular weight polymer. A ratio of [35∶1] was the most efficient at incorporating FGF-2 for both molecular weight species. [B] The loading capacity of complexes was investigated for a [35∶1] ratio of low molecular weight PAGS. This ratio demonstrated a loading efficiency of 50% for all amounts of FGF-2. Statistical significance between [35∶1] and other ratios was noted as “*”, p<0.05.

The loading capacity and efficiency of [PAGS∶heparin] to conjugate FGF-2 were characterized using ^125^I-labeled FGF-2 (^125^I-FGF-2, 1% of loaded growth factor). Loading efficiency was investigated using two different molecular weights of PAGS. The high molecular weight (HMW, M_n_: 73,947, M_w_: 238,802) PAGS showed the highest loading efficiency of 66% (PAGS/heparin ratio is 35) (**Fig. 3a**). It should be noted that FGF-2 incorporation was calculated by measuring the amount of radioactivity present within the sample and not by subtracting residual amount of FGF-2 in supernatant, which would give loading efficiencies 10–20% higher than reported. When the PAGS/heparin ratio was lowered to 21/1 and 7/1 (ratios denote mass ratio unless otherwise noted), the loading efficiency decreased to approximately 59%. Mass ratios lower than 7/1 did not form visible precipitates. A higher mass ratio of 49/1 was attempted but did not improve the loading efficiency (data not shown). The low molecular weight (LMW, M_n_: 63,944, M_w_: 182,023) PAGS yielded a loading efficiency of 50% at the 35/1 PAGS/heparin ratio. LMW PAGS of 21/1 and 7/1 ratios resulted in loading efficiencies of 43% and 40% respectively. The higher molecular weight species of PAGS corresponded to approximately 15% higher loading efficiency at all ratios compared to the lower molecular weight species. The loading capacity of [PAGS∶heparin] complexes was examined using a 35/1 ratio of LMW PAGS (**Fig. 3b**) and the total amount of FGF-2 added was 20, 200, and 2000 ng. In all cases, FGF-2 was loaded with the same efficiency of 50%. This demonstrated that a complex formed by 4 mg PAGS and 115 µg heparin can incorporate at least 1 µg FGF-2, which would be sufficient for most applications if the growth factor bioactivity is maintained.

**Figure 4:**
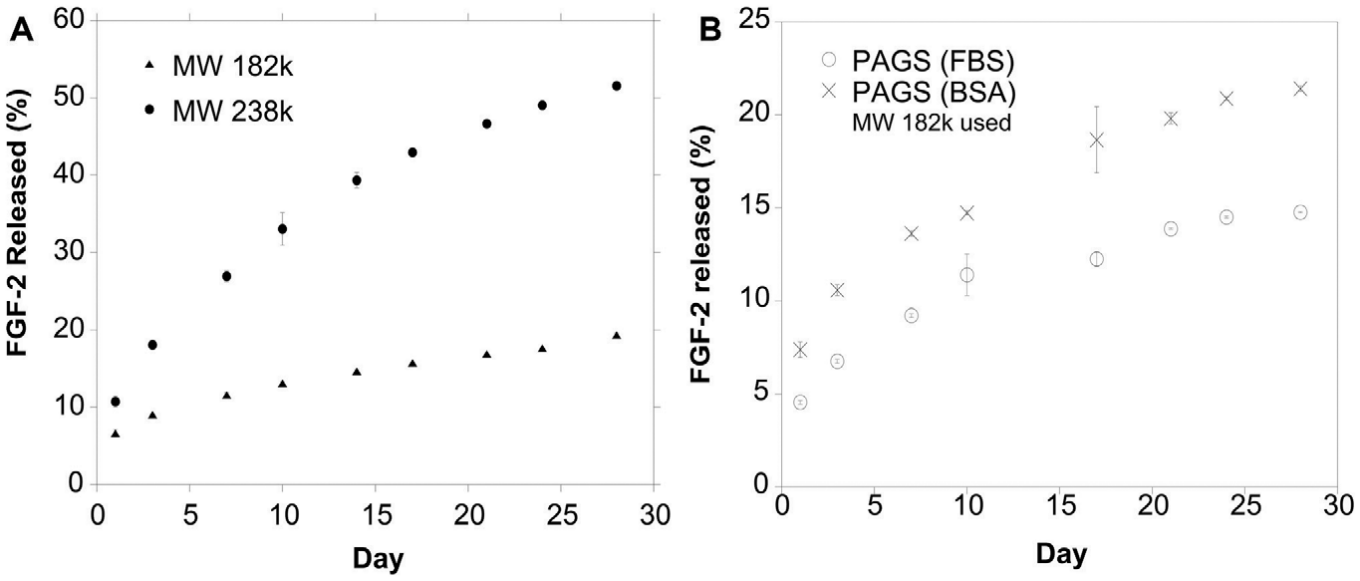
Release kinetics of FGF-2 in PBS and in the presence of serum proteins. [A] Release kinetics as examined by measuring the amount of ^125^I-FGF-2 released from complexes. The percent of FGF-2 released from complexes was monitored over 28 days. Two different molecular weight species of PAGS were used to characterize whether release kinetics were controllable. The LMW species of PAGS released nearly 20% of its loaded growth factor, while the HMW species of PAGS released approximately 50% of incorporated growth factor over the same period of time. [B] The addition of BSA had minor impact on the release FGF-2, which is consistent with the low affinity of BSA with heparin. On the other hand, negatively charged serum proteins in FBS formed precipitate surrounding the delivery matrix, which decreased the FGF-2 release rate.

The release of FGF-2 from the [PAGS∶heparin∶FGF-2] complexes was monitored for 28 days using ^125^I-FGF-2. Two different molecular weight species of PAGS were used to test the hypothesis that increased polyvalency between PAGS and heparin will reduce the affinity of FGF-2 to heparin and increase the release kinetics. The release curves in PBS (**Fig. 4a**) indicated that FGF-2 can be released for at least 28 days. The initial release of approximately 7–10% was similar for both polymers. The release profile of the HMW PAGS approached that of a power law and 52% of the loaded FGF-2 was released over 28 days. If the pattern of the release continues, it should release FGF-2 for up to 110 days. The lower molecular weight species resulted in 19% of loaded FGF-2 released over 28 days. The release also approached that of a power law, and if the trend holds it should release FGF-2 for well over a year. Delivery over a year may not be clinically necessary, but this experiment demonstrated the control, capability, and flexibility of the delivery system. In order to mimic the *in vivo* condition more closely, we conducted the release in the presence of 0.1% bovine albumin (BSA) and 0.5% fetal bovine serum (FBS) [24], [31] using LMW PAGS. The addition of BSA had a minor impact on the release of FGF-2 likely because BSA had a low affinity to heparin (*K*_d_ of 4.3 µM) [36]. The presence of FBS significantly increased the pellet size of the delivery matrix and decreased the release rate of FGF-2. This is most likely caused by the precipitation of serum protein in the presence of residual cations in the [PAGS∶heparin∶FGF-2] complex, which acted as a barrier to the release.

**Figure 5:**
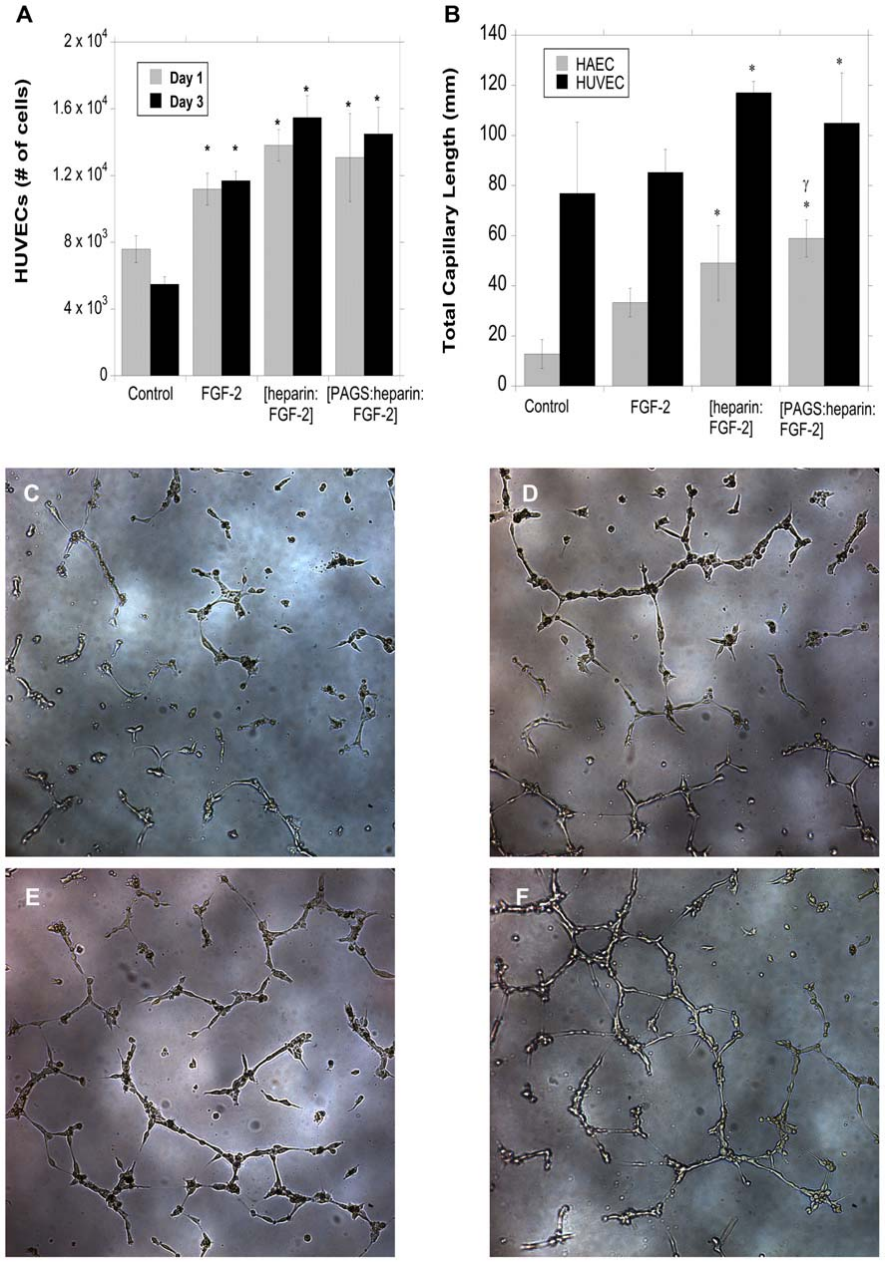
The bioactivity of the FGF-2 released from the [PAGS∶heparin] delviery matrix is higher than bolus FGF-2 and matches that of heparin-protected FGF-2. Note that the growth factor in the release media were 1 and 3 days old, whereas the positive controls were fresh. [A] The mitogenic potency of the released FGF-2 from days 1 and 3 examined using HUVECs indicated that the bioactivity of FGF-2 is well maintained and matched that of the fresh heparin-protected FGF-2. [B] The released FGF-2 demonstrated ability to stimulate endothelial tube formation as well as fresh heparin-protected FGF-2 and is the only group that was statistically significantly better than blous FGF-2 in HAECs. Representative phase contrast micrographs of HUVECs incubated in: [C] no FGF-2, [D] fresh bolus FGF-2, [E] fresh [heparin∶FGF-2], and [F] [PAGS∶heparin∶FGF-2] release media. Statistical significance between the negative control and other experimental groups was noted as “*”, p<0.05. **#** denotes the statistical significance between an experimental group and the bolus FGF-2 group, p<0.05.

The bioactivity of released FGF-2 was investigated by two different assays: endothelial cell proliferation and endothelial tube formation. The ability of FGF-2 to stimulate proliferation of endothelial cells has been well documented. HUVECs were incubated with different groups of media for 48 hours and cell number was measured via a Coulter counter (**Fig. 5a**). The tested media included: [PAGS∶heparin∶FGF-2] release medium, negative control without FGF-2, positive controls of fresh FGF-2 and fresh [heparin∶FGF-2]. HUVECs incubated with [PAGS∶heparin∶FGF-2] day 1 release medium and fresh [FGF-2∶heparin] medium displayed approximately 1.7 fold increase (statistically significant) over the negative control after 48 hours. Conversely, the cell numbers in the bolus FGF-2 and the negative control showed no statistical difference. Although increasing sample size may reveal the difference, this implies the unstable nature of unprotected FGF-2. Data using day 3 release media exhibited the same trend. Thus, the FGF-2 released from [PAGS∶heparin∶FGF-2] complexes was as potent as fresh, heparin-protected FGF-2.

Bioactivity of the released FGF-2 was further analyzed by endothelial tube formation in growth-factor depleted Matrigel®. Both HUVECs and HAECs were cultured with the same group of media as the proliferation assay except that cells were cultured on Matrigel (**Fig. 5b**). Image analysis on total endothelial tube length was measured from a representative image of each well (n = 4) (**Fig. 5c–f** **)**. HAECs incubated with [PAGS∶heparin∶FGF-2] release media exhibited approximately a 4.5 fold increase in tube length compared to the negative control. HUVECs displayed a 1.2 fold increase for the same pair. As observed with the proliferative assay, FGF-2 released from [PAGS∶heparin∶FGF-2] complexes elicited similar cell responses to fresh heparin-stabilized FGF-2 (**Fig. 5b**), and there is no statistical significance between bolus FGF-2 and the negative control. Further, HAECs cultured with [PAGS∶heparin∶FGF-2] media was the only group that stimulated statistically longer tube formation than bolus FGF-2 supplemented media, outperforming fresh heparin-stabilized FGF-2, indictaing the potential of the polycation-based delivery system. The high bioactivity of released FGF-2 is likely attributable to using heparin in its native form.

## Discussion

The [PAGS∶heparin] delivery vehicle takes advantage of heparin's ability to bind and protect numerous growth factors, and mimics the anchoring of growth factors in the ECM. Heparin is known to have a high affinity to FGF-2 (K_d_: 8.6×10^−9^M) [15], which allows the self-assembly of the delivery matrix in aqueous buffer for improved preservation of growth factor bioactivity. Additionally, the delivery matrix is a fluidic suspension that can be injected through a 25 gauge needle enabling minimally invasive delivery. The polyvalent interactions between PAGS and heparin enables the control of FGF-2 release. A high molecular weight PAGS would have a higher polyvalent affinity for heparin, competing more efficiently against FGF-2 and leading to a faster growth factor release. In contrast, a reduced polyvalency between PAGS and heparin would result in a slower release of FGF-2. The molecular weight also impacted the loading efficiency of FGF-2. HMW PAGS is more efficient at conjugating [heparin∶FGF-2] than LMW PAGS. This suggests that the self-assembly of the [PAGS∶heparin∶FGF-2] complex is a kinetically controlled process and the larger polymers with more chain entanglement could precipitate out the [heparin∶FGF-2] complexes more quickly. Refinement of the polymerization and purification process will yield a wider range of PAGS molecular weights, thus expanding the range of the release kinetics to potentially match a large range of clinical applications. This delivery system is expected to be applicable to the large family of heparin-binding growth factors, which will be beneficial for many physiological processes that require multiple growth factors.

## Materials and Methods

### Materials

Succinic acid (TCI, Tokyo, Japan), arginine ethyl ester dihydrochloride (Research Organics, Cleveland, OH), and other reagents (Alfa Aesar, Medford, MA) were used without purification. Meta-chloroperoxy benzoic acid (Acros Organics, Morris Plains, NJ) was lyophilized overnight to remove water on a Labconco FreeZone 2.5 (Kansas City, MO). Flash chromatography was performed on a Buchi Fraction Collector C-660 equipped with a UV photometer C-635 (Flawil, Switzerland). FGF-2 (R&D Systems, Minneapolis, MN) is an N terminal truncated form of human FGF-2 containing the amino acid residues Proline 10 to Serine 155. It is carrier free and reconstituted in PBS with 1mM Dithiothreitol. ^125^I-labeled FGF-2 was purchaced from Perkin Elmer (Waltham, MA). Human umbilical vein endothelial cells (HUVECs) and human aortic endothelial cells (HAECs) were gifts from Dr. Nerem and Dr. Boyan laboratory respectively. Gel permeation chromatography was performed on a GPCmax VE2001 with a 270 Dual Detector (RALS and RI) using a Viscogel I-MBMMW column (Viscotek, Houston, TX). The molecular weight and polydispersity of the polymer are reported relative to polyethylene glycol standards. Scanning electron microscopy (SEM) was performed with a Hitachi S-800 SEM (15 kV, 3–5 nm spot size) or a Leo 1530 SEM (10 kV, 3 nm spot size). Radioactive measurements were measured on a Cobra II Series Auto-Gamma Counter (Perkin Elmer, Waltham, MA). Cell counting was performed on a Multisizer II Coulter Cell Counter (Beckman Coulter, Fullerton, CA). Phase contrast images were captured on a Nikon Eclipse TE2000-U (Melville, NY) equipped with a 4 MP Diagnostics Spot Flex digital camera (Sterling Heights, MI).

### Synthesis of PAGS

The detailed synthesis and biocompatibility study will be reported elsewhere. Briefly, PAGS was synthesized via polycondensation reaction of a 1∶1 molar ratio of diglycidyl succinate and arginine ethyl ester in anhydrous N,N-dimethylforamide under N_2_ at 60°C for 7 days. The resultant polymer was purified and characterized by FTNMR, FTIR, differential scanning calorimetry, and gel permeation chromatography. High Molecular Weight PAGS: M_n_: 73,947, M_w_: 238,802, PDI: 3.22, Low Molecular Weight PAGS: 63,944, M_w_: 182,023, PDI: 2.84.

M_w_: 182,023, PDI: 2.84.

### Preparation of [PAGS∶heparin∶FGF-2] complexes

PAGS (8 mg/ml) and heparin (10 mg/ml) were dissolved in D-PBS with Ca^+2^/Mg^+2^. FGF-2 solution (100 µg/ml) was prepared according to manufacturer's protocol. Heparin and FGF-2 were combined (114 µg/200 ng) and incubated at room temperature (RT) for 15 min under mild agitation on an orbital shaker. PAGS solution (0.5 ml) was added drop-wise to the mixture and allowed to incubate at RT for 30 min with mild agitation. Samples were centrifuged twice for 10 min at 12,100×g. Supernatant was removed and fresh D-PBS was added to the pellet. Each sample contained 4mg PAGS, 114µg heparin, and 200ng FGF-2 (molar ratio: 428/80/1, mass ratio: 17500/500/1). Other ratios of [PAGS∶heparin∶FGF-2] were also used and noted as such.

### Characterization of [PAGS∶heparin∶FGF-2] complexes

[PAGS∶heparin] complexes were prepared as described except that molecular grade water was used. 750 µl of the solution was diluted by half and analyzed using dynamic light scattering and zeta potential measurements. To investigate morphology, [PAGS∶heparin∶FGF-2] complexes were prepared as described except that molecular grade water was used. Otherwise, the large amount of salt will overwhelm the SEM sample. 100 µl of the complex was lyophilized on an SEM aluminum stub and sputtered with gold before SEM examination.

### FGF-2 loading efficiency

[PAGS∶heparin∶FGF-2] complexes were prepared in PBS with 1% of the FGF-2 being ^125^I-labeled FGF-2. After the complex was pelleted via centrifugation, a 5% acetic acid solution in PBS with Ca^+2^/Mg^+2^ was added. The pellet was then agitated overnight to release all the FGF-2 and read on a gamma counter the following day. The amount of FGF-2 loaded was calculated relative to a control of ^125^I- FGF-2 subjected to identical treatments. Mass ratios of [PAGS∶heparin∶FGF-2] examined included 17500/500/1, 10500/500/1, 3500/500/1, 175000/5000/1, and 1750/50/1.

### FGF-2 release kinetics

[PAGS∶heparin∶FGF-2] (mass ratio 17500/500/1) complex with 1% ^125^I-FGF-2 was prepared and pelleted. The supernatant was replaced with 1 ml fresh PBS with Ca^+2^/Mg^+2^ and incubated at 37°C. The complex was centrifuged for 10 min at 12,100×g and the supernatant was analyzed on a gamma counter on 1, 3, 7, 10, 14, 17, 21, 24, and 28 days. 1 ml of fresh PBS was added to replenish the supernatant at each time point. The release kinetics in the presence of protein was performed following the same method except that 0.1 v/v% BSA or 0.5 v/v% FBS was added to the solution and the data were collected on days 1, 3, 7, 10, 17, 21, 24, and 28.

### Mitogenic potency of the released FGF-2

The [PAGS∶heparin∶FGF-2] (heparin/FGF-2 mass ratio, 25/1 [32]) pellets were incubated in 1 ml M-199 medium at 37°C. After 1 and 3 days, the suspension was centrifuged for 10 min at 12,100×g. The supernatant was removed and the tube replenished with 1 ml fresh M-199 medium. HUVECs (P5-10) were seeded in 24-well tissue culture treated polystyrene (TCPS) plates at approximately 10,000 cells/well with 1 ml M-199 growth media. HUVECs were incubated overnight at 37°C, 5% CO_2_, washed with PBS, and incubated with the supernatants from the [PAGS∶heparin∶FGF-2] complexes, fresh FGF-2 of the same concentration (11 ng/ml for day 1 and 7 ng/ml for day 3), fresh heparin-stabilized FGF-2 of the same concentration, and basal medium without FGF-2. The cells were then incubated for another 48 hours before HUVECs were trypsinized and counted on a Coulter counter. The bioactivity of FGF-2 released from [PAGS∶heparin] complexes was determined by comparing cell proliferation with the controls.

### Endothelial tube formation

[PAGS∶heparin∶FGF-2] complexes were pelleted and the supernatant was replaced with 1 ml EGM-2 medium, and incubated at 37°C. After 1 day, the suspension was centrifuged for 10 min at 12,100×g. HAECs (P4-6) and HUVECS (P4-6) were seeded on ECMatrix (Chemicon *In Vitro* Angiogenesis Assay Kit) in a 96-well TCPS plate at 10,000 cells/well. 150 µl supernatant was added to the HAECs culture and incubated for 8h. Endothelial tube formation was quantified by measuring total capillary tube length. Capillary tubes were measured from a representative image of each well using Image Pro. These values were averaged and tested for significance against HAECs incubated with the basal medium, FGF-2- and [heparin∶FGF-2]-supplemented EGM-2 media under identical conditions. HUVEC experiments were carried out in same manner except that M-199 media was used. Each experimental set contained 4 samples.

### Statistics

For each variable group tested there were at least three replicates for the experimental and control samples. Multicomparisons ANOVA, Tukey Method, was used to statistically compare the different experimental values; p<0.05 was considered statistically significant. The results are reported as mean values ± standard deviations.
